# Using natricine snakes to test how prey type and size affect predatory behaviors and performance

**DOI:** 10.3389/fnbeh.2023.1134131

**Published:** 2023-05-05

**Authors:** Noah D. Gripshover, Bruce C. Jayne

**Affiliations:** ^1^Department of Biological Sciences, Florida International University, Miami, FL, United States; ^2^Department of Biological Sciences, University of Cincinnati, Cincinnati, OH, United States

**Keywords:** predator-prey interactions, crayfish-eating snakes, feeding performance, feeding behavior, phylogeny

## Abstract

**Introduction:**

Predation is a complex process for which behavior, morphology, and size of both predator and prey can affect the success and effectiveness of the predator. For predators such as snakes that swallow prey whole, gape ultimately limits prey size, but the behaviors used to select, capture, and consume prey and attributes of the prey can also affect maximal prey size. For example, swallowing live, struggling prey is difficult, but using coiling or envenomation to restrain or kill prey has evolved repeatedly in snakes.

**Methods:**

To test the potential benefits of these behaviors, we manipulated the type and size of prey, and determined how stereotyped predatory behavior was in a snake species (*Liodytes rigida*) that uses both coiling and envenomation to restrain and immobilize its formidable prey of crayfish. We also studied a close relative (*Liodytes pygaea*) that eats fish and salamanders to gain insights into the evolution of these traits.

**Results:**

For *L. rigida*, envenomation of hard-shell crayfish via their soft underside was very stereotyped (100% of feedings). Envenomation of soft-shell crayfish was less frequent (59% of feedings) but became more likely both with increased relative prey size and increased time after molt (hardness). *L. rigida* coiled more for hard-shell than soft-shell crayfish (77% vs. 30%). The probability of coiling was unaffected by prey size, but it increased with increased time after molt for the soft-shell crayfish. *Liodytes rigida* waited to swallow crayfish until they were completely immobile in 75% and 37% of the feedings with hard- and soft-shelled crayfish, respectively. Even with large prey *L. pygaea* never used coiling or envenomation, whereas previous studies of *L. alleni*, the sister species of *L. rigida*, observed non-lethal coiling without envenomation when eating hard-shell crayfish.

**Discussion:**

Our findings for the *Liodytes* clade of three species suggest that coiling evolved ancestral to the crayfish specialists (*L. alleni*; *L. rigida*), and envenomation by *L. rigida* subsequently evolved as an additional means of subduing formidable prey. The proximate benefits observed for coiling and envenomation in *L. rigida* support the evolutionary scenario that both traits evolved to enhance the feeding performance for more formidable prey.

## 1. Introduction

The body plan, size, and behavior of predator and prey can all have important consequences for the success of predators and how they subdue and consume prey (Endler, 1991). For example, body plans with spines, teeth, or claws can impede the ability of predators to subjugate and consume prey (Crofts and Stankowich, 2021) and, in extreme cases, kill a predator (Kornilev et al., 2022). Even when a body plan provides little protection against predators, other attributes of prey can provide defenses such as chemical defenses (Eisner and Meinwald, 1966; Geffeney et al., 2002) and a wide variety of rapid escape behaviors with special neural circuitry such as the Mauthner-initiated C-starts of fishes (Eaton et al., 2001), the tail flips of crayfish (Edwards et al., 1999), and the withdrawal reflex of earthworms (Gunther, 1971). Increased prey size can also increase protection against predators in two major ways. First, increased size is correlated with the ability to generate greater forces that could more effectively deter a predator. Second, size by itself can also prevent predators from eating large prey, if like snakes, the predators do not take bites out of their prey (Price et al., 2015). Large disparities in sizes can even reverse which species is predator or prey as when crocodilians eat juvenile pythons and large pythons eat small crocodilians (Snow et al., 2007).

The more than 3,500 extant species of snakes are all predators (Pough et al., 2016) that have evolved specialized body plans and behaviors that allow them to swallow diverse prey whole (Cundall and Greene, 2000; Cundall and Irish, 2022). Compared to most vertebrates commonly eaten by snakes (Colston et al., 2010; Grundler and Rabosky, 2021), many arthropods, especially crustaceans, have body plans with an armor-like exoskeleton, spiny projections, and powerful claws that impose substantial challenges for predators. Nonetheless, specializing on crustacean prey has evolved in homalopsid snakes (Jayne et al., 2018) and independently in two genera (*Liodytes* and *Regina*) of natricine snakes (Gibbons and Dorcas, 2004). Some of these snakes lack obvious anatomical specializations, but they have evolved behavioral specializations for exploiting formidable crustacean prey (Jayne et al., 2018). Furthermore, some of these species can detect (Jackrel and Reinert, 2011) and exploit crustaceans when they are most vulnerable soon after molting (Jayne et al., 2002). Hence, these taxa are well suited for gaining insights into the primacy of behavior in determining the outcomes of predator-prey interactions, including some profound changes in the risks posed by prey with a given body plan.

After striking their prey, the predatory behaviors of different snake species may vary by: (1) immediately beginning to swallow live, moving prey, (2) rapidly injecting venom and then releasing prey, (3) holding the prey with the jaws during envenomation until the prey is immobilized or dead, or (4) using coils of the body to either restrain or kill prey (Cundall and Greene, 2000). Interestingly, the use of either venom or constriction to kill prey has evolved independently in many different lineages of snakes (Greene and Burghardt, 1978; Palci et al., 2021). This rampant convergent evolution could imply that killing prey before swallowing conveys significant benefits, perhaps including the capacity to consume larger prey. However, empirical data relevant to this plausible suggestion are quite scanty, and approaching this issue by comparing different species of snakes is often complicated by the different types and sizes of prey that are consumed by snake species that also often differ in size. Recently developed methods for quantifying the maximal gape of snakes and cross-sectional area of prey (Jayne et al., 2018, 2022; Gripshover and Jayne, 2021) can reduce some of these difficulties. However, despite the maximal gape of snakes imposing a clear upper limit for prey size, accounting for behavior is vital as snakes may choose prey that are only a small subset of what their gape permits.

In this study we focused mainly on the feeding behavior of *Liodytes rigida* for the following three reasons. First, when eating crayfish this species uses two of the key behaviors, coiling, and envenomation, that are often assumed to facilitate eating large or formidable prey. Second, a prior study (Tumlison and Roberts, 2018) suggested that the predatory behavior of this species is a highly stereotyped fixed action pattern. However, Tumlison and Roberts (2018) only used hard-shell crayfish without methodically manipulating and accounting for prey size, and both of these factors seem likely to affect the risk and difficulty of handling prey. Hence, an open question for this system and many others is whether apparent behavioral stereotypy mainly reflects animals being exposed to a very narrow range of relevant stimuli. Finally, the phylogeny of this species and its relatives is well resolved (Figueroa et al., 2016), and the feeding behavior of *Liodytes alleni*, which is the sister species of *L. rigida*, is well-studied (Gripshover and Jayne, 2021), both of which facilitate gaining insights into the evolution of possible specializations associated with this unusual diet.

We fed *L. rigida*, with known maximal gape, hard- and soft-shell crayfish with a large range in size to test the following two alternative hypotheses regarding predatory behaviors. First, *L. rigida* uses stereotyped feeding behaviors that are unaffected by crayfish size and hardness. Second, plasticity of behavior occurs with changes that are likely to reflect different risks and demands of handling prey. For example, if prey are very small, they are probably less dangerous, and coiling around them may be mechanically more difficult. Additionally, the decreased hardness and mobility of crayfish after molting would be expected to decrease the risk of injury, the difficulty of subduing prey, and the chance of escape, all of which could reduce the probability of both envenomation and coiling. For *Liodytes pygaea*, the sister species of the two crayfish-eating species of *Liodytes*, we offered mostly large vertebrate prey, to determine whether some of the behaviors that facilitate eating crayfish were likely to have been present before the transition from vertebrate to invertebrate prey. Finally, we also compared *L. rigida* to other crustacean specialists including *L*. *alleni* to gain further insights into the potential benefits of evolving specialized behaviors or a venom apparatus.

## 2. Materials and methods

### 2.1. Study species

For laboratory feeding experiments, we collected 10 *Liodytes rigida* and 4 *Liodytes pygaea* from wild populations in Florida, USA (Florida Wildlife Commission permit LSSC-18-00055A). The snout-vent-length (SVL) and masses of these snakes ranged from 140 to 355 mm and 2.1 to 25.2 g for *L. rigida* and from 101 to 275 mm and 0.9 to 13.7 g for *L. pygaea*, respectively. To quantify the scaling relationships of the anatomy of the snakes, we used all the snakes from feeding experiments and some additional individuals to increase sample size (*L*. *rigida*, *n* = 18, SVL = 140–375 mm, mass = 2.1–35.6 g; *L*. *pygaea n* = 11, SVL = 101–295 mm, mass = 0.9–14.0 g).

We fed *L. rigida* crayfish, *Orconectes rusticus*, captured from a wild population in Kenton County, Kentucky, USA. The crayfish were housed individually in transparent containers that allowed recording images every 5 min to determine the time between molting and the beginning of a feeding experiment. We fed *L. pygaea* juveniles of an aquatic, elongate, salamander that lacks hind limbs (*Siren intermedia*) and were captured in the same habitat as the snakes. We also fed *L*. *pygaea* mosquitofish (*Gambusia* spp.) that were obtained from a commercial supplier. We attempted to feed both snake species prey with a wide range of size, including some that seemed likely to be difficult or impossible to eat (Supplementary Table 1).

### 2.2. Anatomical measurements

Immediately after euthanizing snakes with an overdose of isoflurane, we measured their maximal gape using the same procedures that are described in more detail in Gripshover and Jayne (2021). In brief, the probes used to measure gape were cylinders with a hemispherical end that was inserted into the mouth of the snake. We inserted successively larger probes until a probe did not fit or until we observed tissue failure. We then reinserted the next smallest probe that did fit without damaging tissues and recorded its diameter as maximal gape diameter, and we used that to calculate the circular area of maximal gape (Garea). The successive increases in probe diameter were 0.5 and 1.0 mm for diameters less than or greater than 11 mm, respectively.

For all prey we calculated relative prey mass (RPM) and relative prey area (RPA) as the percentages of prey mass and maximal cross-sectional area divided by snake mass and Garea, respectively. To minimize handling and possibly injuring crayfish (especially freshly molted individuals) before they were fed to snakes, we used dorsal view photographs to measure carapace length. We then estimated the cross-sectional area from scaling equations of carapace length and cross-sectional area from a reference collection of preserved crayfish (Gripshover and Jayne, 2021). The cross-sectional area was calculated from an ellipse with axes defined by the maximal height and width of the carapace. For the sirens and fish, we used dorsal and lateral view photographs to obtain the heights and widths needed to calculate the maximal, elliptical cross-sectional areas.

As crayfish harden after molting, the calcium stored in gastroliths is resorbed (McWhinnie, 1962). Thus, to better understand the rate of hardening after molting, we measured the maximal lengths of the two halves of the gastroliths (*n* = 68 individuals preserved 0.3–36 h after molting) and calculated their average, which was converted to a relative size by dividing by the length of the crayfish carapace. We then calculated a linear regression for relative gastrolith size as a function of time after molting.

### 2.3. Experimental procedures

We housed and tested snakes individually in 3.5 L (SVL < 200 mm) or 7.6 L (SVL < 300 mm) plastic pet containers (SVL = 175–233 mm) or a 19 L glass aquarium (SVL = 355 mm) that were underneath 40 W incandescent light bulbs that allowed the snakes to thermoregulate and supplemented the illumination of the lights in the celling of the room during feeding trials. The aquariums were tilted so that a pool of deionized water with a maximum depth of 3 cm covered approximately 75% of the bottom of the aquarium. In aquariums with *L. rigida*, the bottom of the aquarium was covered with a layer of smooth pebbles (Imagitarium River Rock Shallow Creek Gravel, Petco, San Diego, CA, USA), whereas the aquariums with *L. pygaea* had some fragments of aquatic vegetation. We recorded HD (1,920 × 1,080 pixels) videos of all feedings to categorize behaviors and quantify their durations to the nearest second.

The inability to control either the time at which crayfish molted or whether or not a snake would feed did not allow us to standardize either the time between successive feedings or the total number of feedings per individual. The averages for the total number of feedings per individual and the time between successive feedings were 13 and 5.3 days for *L*. *rigida* and 7 and 10.9 days for *L. pygaea*, respectively. Supplementary Table 2 provides additional details regarding replication and the time between successive feedings for individual snakes. When either individual or time between feedings was included in preliminary analyses with univariate and multiple regressions predicting snake behaviors, none of these factors was significant.

The total handling time (HT) was the sum of the durations of the following sequence of six behaviors: (1) attack time extended from the first strike until the final strike that captured prey, (2) envenomation (or holding) occurred when the snakes bit and held the prey without any repositioning or swallowing movements, (3) lateral jaw walking to reposition prey before swallowing, (4) pauses during lateral jaw walking, (5) swallowing time (ST) ended when jaw movements ceased and the prey item was no longer visible, and (6) pauses occurred during swallowing. For *L*. *rigida*, we also recorded the duration of using the body to restrain the crayfish, which commonly overlapped other behaviors following the strike.

We also recorded the number of successful and unsuccessful strikes, the number of escapes by the prey, and the snake and crayfish behaviors preceding capture and when escapes occurred. For the first strike, we recorded whether or not the crayfish was moving at the time of the strike or the time between the last observed movement and the initiation of the strike. When movement occurred it was categorized as: (1) locomotive movements in which the entire body of the crayfish was displaced, (2) appendicular movement without displacement of the entire body, or (3) movement of the antennae. For the final strike, we recorded the longitudinal location of the palate of the snake on the crayfish as follows: (1) posterior half (crayfish abdomen), (2) midbody (the crayfish joint between the abdomen and carapace), (3) anterior half (crayfish carapace), and (4) cheliped of the crayfish. We recorded circumferential locations on the body of prey for both the final strike and the onset of swallowing based on whether the palate of the snake was mainly on the ventral, lateral, or dorsal surface of the prey. We also noted whether the direction of swallowing proceeded starting at the head, midbody, or tail of the prey.

### 2.4. Statistical analysis

To quantify the scaling relationships between gape and the overall size of the snakes, we calculated least squares regressions with log10-transformed data (Kilmer and Rodriguez, 2016). We also used linear regressions to test for significant changes of dependent variables such as duration of behaviors and number of crayfish tail flips with changes in RPA, prey hardness, etc. that were used separately as independent variables. To globally compare feedings on hard-shell and soft-shell crayfish, we categorized crayfish as hard (H_S = 1) or soft (H_S = 0). For some analyses within soft-shell crayfish, we also used the time after molt as a surrogate measure of prey hardness. For the 82 feedings using soft-shell crayfish, the time after molt ranged from 0.12 to 4 h, and the median value was 1.13 h.

We used logistic regressions to test for differences in the likelihood of snakes using a behavior by using the presence of a behavior (0 = absent, 1 = present) as the dependent variable and variables such as RPA or prey hardness as the independent variables. We also used forced-fit multiple regressions to relate independent variables such as RPA and H_S to dependent variables for the duration and presence of different behaviors of the snakes. Our final choice of a multiple regression model was one for which all independent variables had *p*-values < 0.01 and the highest *r*^2^ value was attained, and we report standardized partial regression coefficients to facilitate understanding which independent variable was most important.

We used analyses of covariance (ANCOVA) to compare species, and unless stated otherwise, these comparisons lacked significant heterogeneity of slopes. We performed regression analyses in GraphPad Prism 9 (V 9.4.1) and ANCOVA analyses in R (V 4.2.1).

## 3. Results

### 3.1. Morphology

In both species, maximum gape scaled with negative allometry relative to SVL and mass (Supplementary Table 3). For a given SVL the mass of the two species did not differ significantly, but the maximum gape of *L. rigida* was significantly larger than *L. pygaea* (Supplementary Figure 1 and Supplementary Table 3).

The relative size of the crayfish gastroliths decreased significantly with increased time after molt (*r*^2^ = 0.77, *p* < 0.0001) (Supplementary Figure 2). No gastroliths were visible in any crayfish 29 h after molting and in some individuals as early as 22 h after molting. Thus, after approximately 24 h after molting, the crayfish were likely to be almost fully hardened.

### 3.2. Feeding behavior: *Liodytes rigida*

**FIGURE 1:**
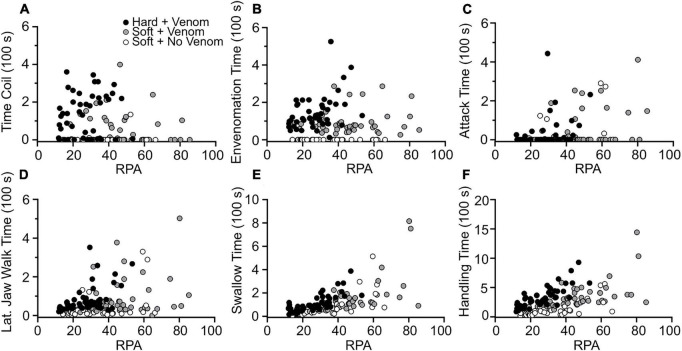
Effects of relative prey size on the duration of feeding behaviors used by *L*. *rigida*. **(A)** Coiling time. **(B)** Envenomation time. **(C)** Attack time. **(D)** Lateral jaw walking time. **(E)** Swallowing time. **(F)** Total handling time.

The *Liodytes rigida* ate 82 soft-shell crayfish (range of relative prey area (RPA) = 14–85%, range of relative prey mass (RPM) = 2–80%) and 47 hard-shell crayfish (RPA = 12–53%, RPM = 2–29%) (Figure 1). The largest value of RPA for soft-shell crayfish eaten (Supplementary Table 1) by each snake (mean = 73%) was significantly greater than that of the hard-shell (mean = 42%) crayfish consumed (paired *t* = 9.73, *df* = 9, two-tailed *p* < 0.0001). Similarly, the largest value of RPA for soft-shell crayfish attacked (mean = 80%) was significantly greater than that of hard-shell (mean = 55%) crayfish attacked (*t* = 3.55, *df* = 9, *p* = 0.006). For hard-shell crayfish, the largest value of RPA for crayfish attacked but not eaten by each snake was significantly greater than that of the crayfish that were eaten (*t* = 4.57, *df* = 9, *p* = 0.001), whereas for the soft-shell crayfish these two prey sizes did not differ significantly (*t* = 1.04, *df* = 9, *p* = 0.28).

The initial strike by the snakes occurred simultaneously with locomotive movements of the crayfish in 83 and 93% of the trials with hard-shell and soft-shell crayfish, respectively, and for hard- and soft-shell crayfish 100 and 93% of the initial strikes were within 2 s of the last movement or during movement of the crayfish, respectively. For the movements that preceded the initial strike, the percentage of trials with locomotive, appendicular, or antennae movements, were 76, 22, and 2% for hard-shell and 73, 25, and 3% for soft-shell crayfish, respectively. Hence, the occurrence of pre-strike movements by crayfish and the rank order of types of movement preceding the strike were very similar despite differences associated with recently molting.

In approximately 10–20% of the feedings, tail flips by the crayfish allowed them to evade a strike (Table 1, pre-capture escape). Tail flips by both soft- and hard-shell crayfish also occurred after capture in more than half the trials, but only approximately one-fifth of these tail flips succeeded in the crayfish escaping (Table 1). For hard-shell crayfish after capture, the likelihood of a tail flip, tail flip with an escape, and pinching, all increased significantly with increased relative prey size (Table 1). For soft-shell crayfish after capture, the likelihood of a tail flip, tail flip with an escape, and pinching all increased significantly with increased time after molt (Table 1). In more than half the feedings with hard-shell crayfish, pinching occurred after capture but resulted in an escape in less than 10% of trials (Table 1). Not only was pinching far less common for soft-shell crayfish, we never observed it resulting in an escape (Table 1).

**TABLE 1:** Percent of feedings on crayfish with behavior.

Behavior	*L. rigida*				L. alleni*	
	Soft no venom (*n* = 34)	Soft venom (*n* = 48)	Soft (*n* = 82)	Hard (*n* = 47)	Soft (*n* = 66)	Hard (*n* = 61)
**Crayfish**						
Pre-capt. tail flip + esc	12	19	16	9	11	16
Post-capt. tail flip	35	75	59^R,H^	53^R^	56	36
Post-capt. tail flip + esc	12	15	13^H^	11^R^	8	0
Post-capt. pinch^H^	9	21	16^H^	55^R^	35	62
Post-capt. pinch + esc	0	0	0	4	2	0
**Snake**						
Cheliped removal	9	8	9^R^	0	0	0
>1 unsuccessful strike	6	6	6^R^	15^R^	12	15
Strike location						
Carapace (C)	29	25	27	28	32	21
C–A joint	47	60	55	51	35	36
Abdomen (A)	18	15	16	19	33	43
Cheliped	6	0	2	2	0	0
Body restraint^H^	12	65	43^R,H^	89	61	79
U-loop	3	10	7	9	18	18
Pin	3	31	20^R^	32^R^	30	25
Coil^H^	6	48	30^H^	77	32	56
Envenomate	0	100	59^R,H^	100	0	0
Crayfish immobile	29	42	37	72	NA	NA
Pre-swallow pause	21	10	15	17	11	13
Swallow pause	15	10	12	13	11	23
Swallow orientation^H^						
Dorsal	15	4	9	2	0	2
Lateral	26	17	21	0	88	92
Ventral	59	79	71	98	12	7
Swallow direction						
Head	21	0	9	0	6	0
Tail	79	100	91	100	94	100

R and H indicate that when the presence (1) or absence (0) of a behavior was a dependent variable in a univariate logistic regression, it changed significantly with increased RPA and prey hardness, respectively (Supplementary Table 5).

*Data are from experiments in Gripshover and Jayne (2021).

**FIGURE 2:**
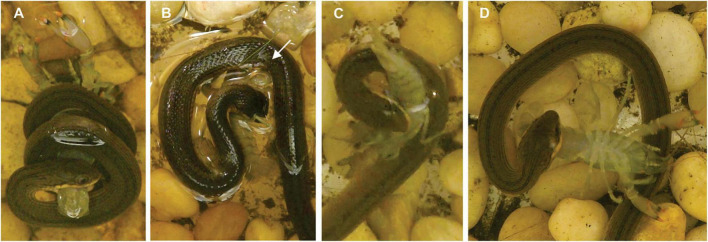
Behaviors used by *L. rigida*. **(A)** Coiling by a snake (SVL = 175 mm, mass = 3.34 g) with a soft-shell crayfish (RPA = 46%, mass = 1.24 g). **(B)** Pinning (beneath the arrow) by a snake (SVL = 185 mm, mass = 3.35 g) with a hard-shell crayfish (RPA = 47%, mass = 0.75 g). **(C)** U-loop followed by envenomation **(D)** by a snake (SVL = 182 mm, mass = 4.00 g) with a soft-shell crayfish (RPA = 39%, mass = 0.84 g).

**FIGURE 3:**
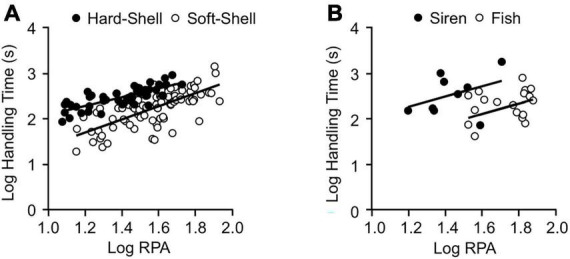
Effects of prey type and relative prey size on the total handling times of *L. rigida* **(A)** and *L*. *pygaea* **(B)**. See Supplementary Tables 5, 7 for regression statistics.

In more than half of all feedings, the snakes struck the joint between the carapace and abdomen (Table 1), and in 90% of the trials snakes struck the dorsal surface of the crayfish. During striking the snakes were very adept at avoiding the claws of crayfish even though the crayfish frequently oriented their body to point their claws toward the snake (Supplementary Video 1). In nearly 90% of the feedings with hard-shell crayfish, the snakes used some form of body restraint on the crayfish after striking it, whereas this happened less than half as frequently for the soft-shell feedings (Table 1). For both hard- and soft-shell crayfish, the forms of body restraint arranged from most to least common were: (1) coiling (Figure 2A), (2) pinning the crayfish under the ventral surface of the snake (Figure 2B), and (3) pushing the crayfish into the lateral, concave surface of part of the snake that formed a U-shaped loop (Figure 2C). In 78% of the 55 trials in which coiling was the first type of body restraint, coiling occurred very rapidly (within 1 s) after the strike, and the mean lag time between the strike and coiling was 1.5 s. In 82% of trials (*n* = 19) with both pinning and coiling, pinning followed coiling. For 9 of the 11 soft-shell crayfish with the longest post-molt times (6–14 h), the snakes used coiling. In the trials with restraint (*n* = 77), the end of restraint was only slightly more likely to occur after (56%) rather than before (46%) the movement of the crayfish ceased.

All the hard-shell and more than half the soft-shell crayfish were envenomated after the strike (Table 1). For all but one of the hard-shell and for 90% of the soft-shell crayfish, envenomation occurred by the snake repositioning its mouth and then holding it so that the palate contacted the soft underside of the crayfish abdomen (Figure 2D and Supplementary Video 1). The snakes commonly bit the crayfish repeatedly as they held the crayfish with their jaws and presumably envenomated them. Some form of body restraint was used in 77% of the trials with envenomation, and the form of restraint was coiling in 81% of the subset of cases with both restraint and envenomation. The lag time between the onset of coiling and the onset of envenomation ranged from 4 to 98 s and averaged 30 s (*n* = 55), which seems likely to provide ample time for the snakes to respond to feedback regarding the prey item.

Before swallowing began, the crayfish were immobile in nearly three-fourths of the hard-shell feedings but only approximately half that amount for the soft-shell feedings (Table 1). Hard-shell crayfish were swallowed tail first and ventral side up in all and all but one of the feedings, respectively (Supplementary Video 1), and these were also the most common but less frequent swallowing orientations for the soft-shell crayfish (Table 1). The snakes paused during swallowing in approximately 10% of all trials.

**FIGURE 4:**
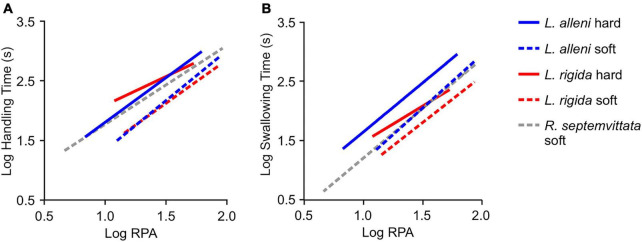
Effects of prey type and relative prey size on the total handling time **(A)** and swallowing time **(B)** for three species of natricines that consume crayfish. Supplementary Tables 4, 5 for regression statistics.

Univariate regressions using either RPA or the molt condition (H_S) of the crayfish as the independent variable revealed many significant effects on the occurrence and duration of many aspects of feeding behavior, but many of these relationships explained little (*r*^2^ < 0.10) of the variation in the dependent variable (Supplementary Table 4). For the total of 129 feedings, three of the clearest trends, arising when the crayfish were hard rather than soft, were more bites during envenomation (*r*^2^ = 0.45) and increases in the probability of performing any form of body restraint (*r*^2^ = 0.21) or coiling (*r*^2^ = 0.19). Some noteworthy trends for the 82 feedings on soft-shell crayfish were that as RPA increased, significant increases occurred for swallowing time (*r*^2^ = 63%), total handling time (*r*^2^ = 0.51), attack time (*r*^2^ = 0.47), and the number of tail flips (*r*^2^ = 0.22). Crayfish being hard- rather than soft-shell increased the number of pinches (*r*^2^ = 0.25), the lateral jaw walking time (*r*^2^ = 0.15), and the total handling time (*r*^2^ = 0.13). For the 47 feedings on hard-shell crayfish, increased RPA also increased total handling time (*r*^2^ = 0.59), swallowing time (*r*^2^ = 0.55) and lateral jaw walking time (*r*^2^ = 0.37). Assuming the slopes met the assumption of homogeneity, an ANCOVA with RPA as a covariate (Supplementary Table 5) revealed significantly shorter total prey handling times for soft- compared to hard-shell crayfish (Figures 3A, 4A). For the hard-shell crayfish, envenomation time increased with increased RPA (*r*^2^ = 0.16). However, for the 48 soft-shell crayfish that were envenomated, the envenomation time did not change significantly either with increased RPA (*r*^2^ < 0.01) or increased hardness (*r*^2^ = 0.04), and the mean value of 93 s was less than 70% of the values observed for hard-shell crayfish (Figure 1B). Hence, the snakes appear to modulate the amount of venom in response to the interactive effects of RPA and molt status of the crayfish.

In a multiple regression with data combined for soft- and hard-shell feedings, prey handling time increased significantly (*r*^2^ = 0.71) with increased RPA, the presence of envenomation and for hard- rather than soft-shell crayfish (Supplementary Table 6). Additionally, swallowing times increased significantly with increased values of RPA, for hard- rather than soft-shell crayfish and the presence of coiling (*r*^2^ = 0.65), crayfish escapes via tail flips after capture, and the presence of snakes coiling. A multiple regression (*r*^2^ = 0.30) revealed that the presence of coiling was significantly more likely for hard vs. soft-shell prey and when envenomation was present (Supplementary Table 6). For the presence of envenomation, no pair of independent variables in a logistic multiple regression were both individually significant and with a higher *r*^2^ than a regression using only the presence of coiling as an independent variable (*r*^2^ = 0.25).

### 3.3. Feeding behavior: *Liodytes pygaea*

The four *L. pygaea* ate a total of nine sirens (RPA = 16–52%, RPM = 9–35%) and 18 fish (RPA = 34–104%, RPM = 4–21%) (Figure 3B and Supplementary Table 1). All the snakes attacked the largest sirens offered (RPA = 56, 45, 41, and 39%), but only one (RPA = 39%) succeeded in eating the largest siren attacked. All the snakes attacked and succeeded in eating the largest fish offered (RPA = 104, 101, 96, and 74%). Unlike *L. rigida*, we never observed *L. pygaea* using its body to restrain prey nor did we find any evidence of envenomation. The snakes most commonly struck the fish on the head, but the snakes were equally likely to strike the sirens on the head or near midbody (Table 2). Prey commonly (>30% of trials) escaped from the snakes (Table 2). For sirens, pauses were most common prior to swallowing, whereas for fish, pauses were most common during swallowing (Table 2). The duration of pre-swallow pausing increased significantly with increased RPA (Supplementary Table 7). The snakes commonly paused during repositioning (Table 2), especially if the prey began to move conspicuously. Both fish and sirens were usually swallowed head first (Table 2). A multiple regression revealed that values of HT for *L. pygaea* increased significantly for increased values of RPA and for sirens compared to fish (Figure 3B and Supplementary Table 6).

**TABLE 2:** Percent of feedings of *L*. *pygaea* with behavior.

Behavior	Percent of feedings with behavior	
	Siren (*n* = 9)	Fish (*n* = 18)
**Prey**		
Pre-capture escape	33	39
Post-capture escape	11	33
**Snake**		
Strike location^R^		
Head	44	78
Midbody	44	6
Tail	11	17
Body restraint	0	0
Envenomate/Hold	0	0
Pre-swallow pause	67	22
Swallow pause	33	61
Prey orientation at swallow		
Dorsal	0	28
Lateral	100	67
Ventral	0	6
Direction swallow		
Head^R^	56	83
Midbody	11	0
Tail	33	17

R indicates that when the presence (1) or absence (0) of a behavior was a dependent variable in a univariate regression, it changed significantly with increased RPA (Supplementary Table 7).

## 4. Discussion

Our results emphasize how size and other attributes of prey can profoundly affect the behaviors used by a predator to capture and consume prey. For hard-shell crayfish, the occurrence of envenomation and swallowing prey from tail to head were indeed highly stereotyped for *L*. *rigida* as previously reported (Tumlison and Roberts, 2018). Nonetheless, many other aspects of predatory behavior varied substantially with variation in prey; hence, we evaluate whether any evidence suggests that decreased stereotypy in the predatory behavior of *L*. *rigida* is associated with attributes of prey that likely make them easier to subdue and consume. Two key issues are what stimuli elicit specialized behaviors and what, if any, benefits do those specialized behaviors convey. Additionally, we use variation within a species as well as comparative data to gain general insights into the evolution of specializations, or the lack thereof, for gape-limited predators that exploit unusual or formidable prey.

The variable use of envenomation and coiling by *L*. *rigida* provides one line of evidence for testing the potential benefits of body restraint and envenomation. The nearly universal use of both envenomation and coiling with hard-shell crayfish is consistent with these behaviors being elicited by attributes of prey that make them more difficult to handle. However, unexpectedly, the global effect of envenomation was to increase rather than decrease total handling time because the envenomation was often a sizeable fraction of total handling time (mean = 37%; range 6–84%). Swallowing time, which excludes envenomation time, increases significantly with increased RPA and for hard- versus soft-shell crayfish (Figure 4B), but we could not detect a significant benefit (negative effect) of envenomation on swallowing time within *L*. *rigida*. Within the soft-shell crayfish, envenomation was slightly more likely to occur with increased values of RPA and hardness, and it was associated with the occurrence of post-capture tail flips. Thus, despite increased size, hardness, and movement eliciting envenomation, the benefits of envenomation for handling prey are subtle at best based only on variation within our study species.

Comparing *Liodytes rigida* and its sister species *L*. *alleni* provides additional insights into the potential benefits of envenomation because they both eat crayfish and use body restraint, but only *L*. *rigida* uses envenomation (Franz, 1977; Tumlison and Roberts, 2018; Gripshover and Jayne, 2021). Unexpectedly, compared to *L*. *alleni*, *L*. *rigida* did not have a lower incidence of post-capture escapes, and the probabilities of post-capture pinching occurring and the total handling times were quite similar for a given size and type of the crayfish (Figure 4A and Table 1). Unlike *L*. *alleni, L*. *rigida* almost releases the crayfish while repositioning its jaws to envenomate the crayfish, and the teeth of *L*. *rigida* lack the highly specialized shape in *L*. *alleni* that is believed to facilitate holding hard prey (Rossman, 1963). Perhaps, both of these factors contribute to a slightly lower chance of crayfish escaping *L*. *alleni*. However, faster swallowing times of *L*. *rigida* compared to *L*. *alleni* (Figure 4B and Supplementary Table 5) provide some strong evidence of at least one benefit of envenomation by *L*. *rigida*. Interestingly, the processes of subjugation and consumption (Endler, 1991) are decoupled when *L*. *rigida* (or any other predator) kills its prey, whereas these processes are inseparably coupled in *L*. *alleni* and a host of other snake species that swallow their prey live. Perhaps envenomation by *L*. *rigida* also reduces the chance of internal injury, but we lack the data needed to address this.

**FIGURE 5:**
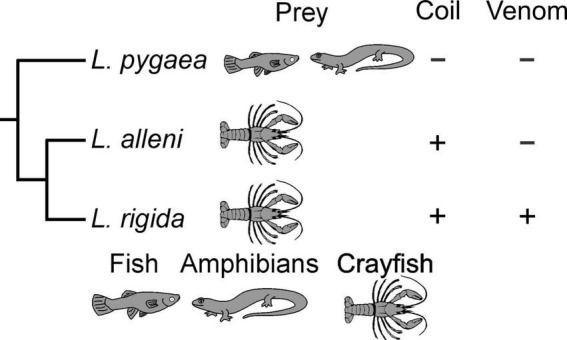
Phylogenetic summary of diet and feeding behaviors for snakes in the genus *Liodytes*. The presence of prey is notated by illustrations and the presence or absence of a behavior is notated as (+) or (–), respectively. Phylogeny is based on Figueroa et al. (2016), and in this rendition the branch lengths are arbitrary.

The evolutionary origin and benefits of *L*. *rigida* using its body to restrain prey become more apparent with comparisons of other natricine species. Body restraint of prey is quite rare in the clade of North American natricines (Rossman et al., 1996; Gibbons and Dorcas, 2004), which includes three major lineages with the following genera: (1) *Liodytes* and their relatives, (2) *Thamnophis*, and (3) *Nerodia* and *Regina* (Figueroa et al., 2016). The widespread lack of body restraint implies this was the ancestral condition for this entire clade and was retained in *L*. *pygaea*. Hence, body restraint probably evolved in the common ancestor of *L*. *rigida* and *L*. *alleni* and was closely associated with another derived trait of a specialized diet of hard-shell crayfish (Figure 5). *Regina septemvittata* eats only soft-shelled crayfish, and they do so without any body restraint although they often hold their prey with their jaws for a substantial time before swallowing commences (Gripshover and Jayne, 2021). For soft-shell crayfish, the faster handling times of both *L*. *rigida* and *L*. *alleni* compared to *R*. *septemvittata* (Figure 4A) provide strong evidence for a benefit of body restraint for feeding performance on a given type and size of prey.

Similar to the rarity of body restraint within North American natricines, well documented cases of envenomation of prey by these snakes are also quite rare (Rossman et al., 1996; Gibbons and Dorcas, 2004) although several species of old world natricines commonly use envenomation (Weinstein et al., 2011). The absence of envenomation in both *L*. *pygaea* and *L*. *alleni* suggests that rather than being retained from a common ancestor of *Liodytes*, the capacity for envenomation evolved in *L*. *rigida* (Figure 5).

Unlike the independent evolutionary origins of a specialized crustacean diet within the North American natricines, all three of the genera of Southeast Asian homalopsid snakes that specialize on crustaceans form a monophyletic group (Figueroa et al., 2016). All three of these genera have retained the opisthoglyphous venom apparatus (Jayne et al., 2018) that occurs in most homalopsid species and is often used to kill vertebrate prey (Jayne et al., 1988; Murphy, 2007). Unlike *L*. *rigida* and rather paradoxically, all three of the crustacean-eating homalopsids have sizeable, grooved rear fangs, but they do not appear to envenomate their prey. Unlike crayfish, the crabs eaten by homalopsids have both ventral and dorsal surfaces that are heavily armored and likely to prevent penetration by the teeth of the snakes. Presumably teeth could penetrate soft-shell crabs, but neither *Fordonia leucobalia* nor *Gerarda prevostiana* appear to envenomate these prey (Jayne et al., 2018). *Cantoria violacea* eats snapping shrimp, which have a soft underside similar to crayfish, but envenomation appears absent for these prey which are usually swallowed dorsal side up (Jayne et al., 2018). The venoms of snakes can have considerable specificity as indicated by some that are highly effective for reptiles but not mammals (Mackessy et al., 2006) or more toxic for arthropods without increased toxicity for vertebrates (Starkov et al., 2007). Thus, an interesting issue to resolve in the crustacean-eating snakes is the importance of venom composition versus a mechanical barrier in prey for impeding successful envenomation.

In common with *L*. *rigida* and *L. alleni*, all three of the crustacean-eating homalopsid species use some form of body restraint (Jayne et al., 2018). *Cantoria violacea* occasionally uses its ventral surface to pin snapping shrimp. *Fordonia leucobalia* strikes both hard- and soft-shell crabs with a closed mouth and then uses its chin to pin crabs before quickly coiling around them. Hence, the use of body restraint in *F*. *leucobalia* is more stereotyped than *L*. *rigida* as the latter species commonly lacked body restraint for soft-shelled prey. *Gerarda prevostiana* only eats soft-shell crabs, and with increased prey size it relies increasingly on coiling to restrain prey to facilitate ripping them apart, which is unlike the generalized prey handling behaviors of the two species of *Regina* that feed exclusively on soft-shell crayfish. None of these three species that specialize on soft-shell crustaceans have any obvious morphological specializations, whereas two of the species specializing on hard-shell crustaceans (*L*. *alleni* and *F*. *leucobalia*) have very unusual, specialized dentition that appears better suited than generalized tooth morphology for coping with hard surfaces (Rossman, 1963; Savitzky, 1983). Furthermore, all four of the species specializing on hard-shell crustaceans (*L*. *rigida*, *L*. *alleni*, *C*. *violacea*, and *F*. *leucobalia*) use at least one specialized behavior for handling prey. Collectively, this system reinforces the pivotal role that behavioral innovations can have for exploiting novel, formidable prey even without accompanying anatomical specializations. In addition, the intuitively appealing evolutionary scenario that snakes ate less dangerous soft-shell crustaceans before more dangerous hard shell-prey is not supported either for the homalopsids or for the two species of *Liodytes* that eat hard-shell crustaceans.

In some lineages of snakes, especially those that kill prey by constriction rather than just coiling to restrain prey, the pattern of coiling and the use of coiling can be quite stereotyped (Greene and Burghardt, 1978). By contrast, this study and a previous study (Gripshover and Jayne, 2021) found both species of *Liodytes* that eat crayfish use their bodies in variable ways to restrain prey even though the occurrence of body restraint was quite stereotyped for hard-shell crayfish. Perhaps killing prey with coiling is functionally more demanding than merely restraining prey, and that could be a significant factor contributing to stereotypy in many constricting snakes.

As the speed of predatory behaviors increases, one might expect that the ability to incorporate feedback to modify the behavior would diminish. Although it is not as fast as some other vertebrate predatory behaviors, such as suction feeding of fish or tongue projection by plethodontid salamanders, the duration of many snake strikes still appears short enough to afford little opportunity to incorporate feedback to modify the strike after it is initiated (Cundall et al., 2007). However, pre-strike information regarding the location, type, and size of prey could allow snakes to use different preset motor programs that would not otherwise be subject to feedback (Cundall et al., 2007). The refusal of *L*. *rigida* to attack very large prey (even when they were smaller than maximal gape) and the high accuracy of strike location suggest visual cues are important for such pre-attack modulation of behavior. The high percentages of initial strikes that were associated with prey movement further support the use of visual cues by *L*. *rigida*, and visual cues are known to be sufficient to elicit attacks in several other species of natricine snakes (Drummond, 1985). Prey moving in water also generate vibrations, and vibrations elicit attacks in at least some species of aquatic snakes (Jayne et al., 1988; Catania et al., 2010). Hence, the common defensive behavior of remaining immobile (Nishiumi and Mori, 2015; Roelofs, 2017) could facilitate crayfish evading detection by *L*. *rigida*, and we observed several trials in which this appeared to be happening. Since some natricines can use olfaction to detect freshly molted crayfish (Jackrel and Reinert, 2011), *L*. *rigida* could also know the molt condition of prey prior to attack. Olfactory cues and vibrations seem likely to have additive effects with visual cues for increasing the probability of attacks by snakes on aquatic prey (Drummond, 1985). Once *L*. *rigida* contacts prey, a variety of tactile cues from the variable hardness and the vigor of prey movements could provide additional feedback for modifying the behaviors of coiling and envenomation, both of which are much slower than the strike.

The benefits of highly toxic venoms for subduing prey seem obvious for the elapid and viperid snakes with hollow fangs at the front of their mouth through which venom can be forcibly (Mackessy and Saviola, 2016) and rapidly (<200 ms) injected (Cundall, 2009). However, much remains to be understood regarding the benefits of a more rudimentary venom apparatus as occurs in *L*. *rigida* and a wide-variety of rear-fanged snakes (Weinstein et al., 2010, 2011; Mackessy and Saviola, 2016). The morphology of front-fanged snakes could permit modulating the amount of venom delivered within a fraction of a second (Cundall, 2009), whereas for snakes such as *L*. *rigida* without hollow fangs, the primary mechanism for modulating the amount of venom delivered seems likely to be how long they bite and hold their prey, which can take several minutes. Interestingly, many of these snakes lacking hollow fangs also use their body to restrain or constrict prey (Rochelle and Kardong, 1993); hence, they have some redundancy for how prey are subdued. An interesting issue for these snakes is how commonly the evolution of body restraint may have preceded venom apparatus or enhances the utility of rudimentary venom apparatus by securing prey during the prolonged process of venom delivery.

In part because of their medical importance, envenomation by viperids and elapids is better studied compared to rear-fanged snakes (Weinstein et al., 2011). However, the diverse diets, variable toxicity and specificity of venom, frequent use of body restraint and constriction, and the phylogenetic diversity of rear-fanged snakes provide a rich, model system for future study of the evolution of specialized morphologies and behaviors involved in predator prey interactions. To a great extent *L*. *rigida* used envenomation and body restraint as needed resembling many species of venomous snakes that apparently use their venom frugally (Morgenstern and King, 2013), which would require some sensory information or feedback regarding prey. However, an alternative strategy could be to be minimally reliant on feedback and use “overkill” to maximize the speed of subduing prey and minimize the risk to the predator. Testing between these two alternatives or for a continuum of variation between them may be another fruitful area for future comparative studies, especially in light of the tremendous variability in the times for which venom apparatus and specialized predatory behaviors have evolved in different lineages of snakes.

## Data availability statement

The original contributions presented in this study are included in the article/Supplementary material, further inquiries can be directed to the corresponding author.

## Ethics statement

This animal study was reviewed and approved by the Institutional Animal Care Use Committee of the University of Cincinnati (protocol number: 07-01-08-01).

## Author contributions

Both authors contributed to the all aspects of the conceptualization, methodology, investigation, data analysis, and writing and article and approved the submitted version.
