# Supplementary material for: Using natricine snakes to test how prey type and size affect predatory behaviors and performance
Source: Front Behav Neurosci. 2023 May 5;17:1134131. doi: 10.3389/fnbeh.2023.1134131 (PMC10196387; doi:10.3389/fnbeh.2023.1134131)
Supplement: Supplementary file 1 [file Data_Sheet_1.pdf]

Supplementary Material

Using natricine snakes to test how prey type and size affect predatory behaviors and performance

Noah D. Gripshover*, Bruce C. Jayne

*** Correspondence**: Noah D Gripshover: ngrip001@fiu.edu

1 Supplementary Tables

Table S1 | Maximal values of RPA (%) for prey offered, attacked and consumed by each snake.

**Individual**	**Soft-shell crayfish**			**Hard-shell crayfish**		
L. rigida	**Offered**	**Attacked**	**Consumed**	**Offered**	**Attacked**	**Consumed**
1	66	66	66	77	46	40
2	79	79	79	58	58	35
3	82	82	82	51	51	45
4	82	82	82	52	50	44
5	118	73	73	61	61	45
6	95	68	68	61	61	50
7	121	121	55	50	50	39
8	86	86	85	51	51	44
9	63	63	63	53	37	25
10	76	76	75	95	88	53
L. alleni*						
1		89	71		48	32
2		84	55		39	27
3		56	56		27	27
4		66	66		61	44
5		65	65		42	42
6		90	75		67	67
7		98	80		63	63
8		120	87		58	47
9		94	89		62	45
10		40	40		32	24
L. pygaea	**Siren**			**Fish**		
1	56	56	52	101	101	80
2	44	44	22	74	74	74
3	39	39	39	104	104	104
4	41	41	40	96	96	96

*Data are from experiments in Gripshover and Jayne (2021).

Table S2 | The number of feedings for each snake and the average number of days between feedings

Snake Number	Number of Feedings	Prey Type 1	Prey Type 2	Average (S.E.M) Interval Between Feedings
L. rigida		**Hard**	**Soft**	
1	6	1	5	7.2 (2.2)
2	9	4	5	4.1 (1.5)
3	12	5	7	7.2 (1.1)
4	12	4	8	3.4 (0.9)
5	12	5	7	3.3 (1.2)
**6**	14	6	8	6.2 (1.4)
7	14	5	9	5.3 (1.1)
8	15	5	10	5.4 (1.8)
9	17	6	11	6.2 (2.0)
10	18	6	12	5.2 (1.2)
L. pygaea		**Fish**	**Siren**	
1	5	3	2	15.0 (5.2)
2	6	3	3	12.2 (5.2)
3	7	6	1	9.0 (3.8)
4	9	6	3	7.3 (1.3)

TABLE S3 | Least squares scaling relationships of snake of morphology.

Independent variable	Dependent Variable	Slope ± 95% CL	Intercept ± 95% CL	*n*	r^2	*p*
L. rigida						
Log SVL (mm)	log Mass (g)	2.873 ± 0.207	-5.886 ± 0.483	18	0.98	<0.0001
Log SVL (mm)	log Diam (mm)	0.671 ± 0.129	-0.512 ± 0.300	18	0.88	<0.0001
Log SVL (mm)	log Area (mm^2)	1.342 ± 0.257	-1.129 ± 0.600	18	0.88	<0.0001
Log Mass (g)	log Diam (mm)	0.227 ± 0.050	0.867 ± 0.045	18	0.85	<0.0001
Log Mass (g)	log Area (mm^2)	0.454 ± 0.100	1.629 ± 0.091	18	0.85	<0.0001
L. pygaea						
Log SVL (mm)	log Mass (g)	2.783 ± 0.188	-5.649 ± 0.412	11	0.99	<0.0001
Log SVL (mm)	log Diam (mm)	0.747 ± 0.153	-0.757 ± 0.334	11	0.92	<0.0001
Log SVL (mm)	log Area (mm^2)	1.493 ± 0.306	-1.619 ± 0.669	11	0.92	<0.0001
Log Mass (g)	log Diam (mm)	0.267 ± 0.054	0.759 ± 0.036	11	0.93	<0.0001
Log Mass (g)	log Area (mm^2)	0.535 ± 0.108	1.412 ± 0.071	11	0.93	<0.0001

TABLE S4 │ Univariate regressions predicting handling times and behaviors for *L. rigida*.

Independent Variable	Dependent Variable	Slope ± 95% CL	Intercept	r^2	*p*
**all (*n* = 129)**					
H_S	Number of Bites	5.224 ± 1.006	2.244 ± 0.610	0.45	<0.0001
H_S	Orientation Swallow	-0.336 ± 0.196	1.378 ± 0.118	0.08	0.0009
H_S	Log Envenom. Time	0.160 ± 0.114	1.898 ± 0.080	0.08	0.0063
H_S	If Prey Restraint	2.423 ± 1.140	-0.295 ± 0.444	0.21	<0.0001
H_S	If Coil Crayfish	2.010 ± 0.77	-0.824 ± 0.480	0.19	<0.0001
H_S	If Movement Ceased	1.511 ± 0.809	-0.550 ± 0.460	0.11	0.0012
H_S	If Post-Capture Pinch	1.883 ± 0.853	-1.669 ± 0.638	0.15	<0.0001
**soft-shelled (n= 82)**					
Log RPA	Num Strikes	1.852 ± 1.599	-1.211 ± 2.538	0.06	0.0238
Log RPA	# Post-Capt Tail Flip	6.434 ± 2.693	-8.187 ± 4.273	0.22	<0.0001
Log RPA	Num Prey Restraint	0.970 ± 0.851	-0.955 ± 1.351	0.06	0.0261
Log RPA	Num Bites	5.306 ± 2.378	-6.117 ± 3.774	0.20	<0.0001
Log RPA	Log Attack Time	2.451 ± 1.126	-2.215 ± 1.831	0.47	<0.0001
Log RPA	Log Jaw Walk Time	1.212 ± 0.538	-0.373 ± 0.852	0.20	<0.0001
Log RPA	Log Swallow Time	1.572 ± 0.269	-0.553 ± 0.423	0.63	<0.0001
Log RPA	Log Handling Time	1.460 ± 0.321	-0.060 ± 0.510	0.51	<0.0001
Prey Hardness	Num. Pinch	0.125 ± 0.048	-0.045 ± 0.184	0.25	<0.0001
Prey Hardness	Num. Prey Restraint	0.089 ± 0.054	0.349 ± 0.208	0.12	0.0016
Prey Hardness	Num. Bites	0.204 ± 0.170	1.730 ± 0.647	0.07	0.0186
Prey Hardness	Log Lat Jaw Walk Time	0.068 ± 0.037	1.365 ± 0.141	0.15	0.0004
Prey Hardness	Log Swallow Time	0.030 ± 0.028	1.851 ± 0.108	0.05	0.0406
Prey Hardness	Log Handling Time	2.117 ± 0.108	0.049 ± 0.028	0.13	0.0009
Log RPA[1]	If Cheliped Removal	7.150 ± 7.005	-14.27 ± 12.37	0.09	0.0216
Log RPA[1]	If Pin Prey	5.442 ± 4.305	-10.28 ± 7.010	0.11	0.0060
Log RPA[1]	If Envenomate	4.346 ± 2.569	-6.463 ± 4.577	0.13	0.0017
Log RPA[1]	If Post-Capt Tail Flip	5.508 ± 3.228	-8.276 ± 5.064	0.19	0.0003
Prey Hardness[1]	If Coil Crayfish	0.334 ± 0.221	-1.734 ± 0.796	0.16	0.0010
Prey Hardness[1]	If Envenomate	0.230 ± 0.243	-0.172 ± 0.626	0.07	0.0332
Prey Hardness[1]	If Post-Capt. Tail Esp	0.192 ± 0.189	-2.463 ± 1.044	0.05	0.0450
Prey Hardness[1]	If Post-Capt. Tail Flip	0.314 ± 0.289	-0.329 ± 0.655	0.11	0.0128
Prey Hardness[1]	If Post-Capt. Pinch	0.371 ± 0.229	-2.925 ± 1.201	0.17	0.0005
**hard-shelled (n=47)**					
Log RPA	Num. Strike	2.095 ± 1.390	-1.388 ± 1.954	0.17	0.0040
Log RPA	Post-Capt. Tail Esp	0.561 ± 0.498	-0.676 ± 0.699	0.10	0.0280
Log RPA	Post-Capt. Tail Flip	4.349 ± 2.427	-4.700 ± 3.410	0.22	0.0008
Log RPA	Post-Capt. Pinch	0.394 ± 0.323	-0.506 ± 0.454	0.12	0.0181
Log RPA	Num. Bites	5.838 ± 5.612	-0.669 ± 7.890	0.08	0.0419
Log RPA	Log Prey Restr. Time	0.588 ± 0.530	1.375 ± 0.744	0.11	0.0305
Log RPA	Log Enven. Time	0.699 ± 0.490	1.084 ± 0.688	0.16	0.0061
Log RPA	Log Lat Jaw Walk Time	1.041 ± 0.411	0.346 ± 0.578	0.37	<0.0001
Log RPA	Log Swallow Time	1.216 ± 0.332	0.260 ± 0.467	0.55	<0.0001
Log RPA	Log Handling Time	0.966 ± 0.241	1.130 ± 0.338	0.59	<0.0001
Log RPA[1]	If >1 Strike	5.491 ± 4.99	-8.467 ± 7.353	0.15	0.0147
Log RPA[1]	If Pin Prey	4.439 ± 4.663	-7.056 ± 6.844	0.10	0.0372
Log RPA[1]	If Post-Capt. Tail Flip	7.204 ± 5.066	-9.909 ± 7.151	0.25	0.0016
Log RPA[1]	If Post-Capt. Pinch	3.710 ± 3.839	-4.942 ± 5.368	0.09	0.0422

[1]Logistic regression used to predict binomial dependent variable, we calculated the r^2 using the Cox-Snell's method. For soft-and hard crayfish, H_S = 0 and 1, respectively.

TABLE S5 | ANCOVA results comparing species of *Liodytes* and hard vs. soft crayfish.

Factor	Covariate (crayfish subsample)	Dependent Variable	Effect	Factor F_{df} (p)	Covariate X Factor Interaction F_{df} (p)
Species	Log Mass	Log SVL	Lr = Lp	$2.2_{1,26}$ (0.152)	$0.47_{1,25}$ (0.50)
Species	Log Garea	Log SVL	Lr > Lp	$21.3_{1,26}$ (<0.0001)	$0.72_{1,25}$ (0.41)
Species	Log Garea	Log Mass	Lr > Lp	$23.9_{1,26}$ (<0.0001)	$1.4_{1,25}$ (0.244)
H_S	Log RPA (Lr)	Log HT	H > S	$93.2_{1,126}$(<0.0001)	$4.2_{1,25}$ (0.043)
Species	Log RPA (S)	Log ST	Lr < La	$46.8_{1,145}$ (<0.0001)	$1.31_{1,144}$ (0.254)
Species	Log RPA (S)	Log HT	Lr = La	$2.3_{1,145}$ (0.132)	$0.94_{1,144}$ (0.333)
Species	Log RPA (H)	Log ST	Lr < La	$55.8_{1,105}$(<0.0001)	$3.4_{1,104}$(0.070)
Species	Log RPA (H)	Log HT	Lr = La	$2.6_{1,105}$ (0.112)	$6.1_{1,104}$ (0.015)
Species	Log Garea	Log SVL		N/A	$7.8_{1,43}$ (0.008)
Species	Log Garea	Log Mass	Lr = La	$0.57_{1,44}$ (0.453)	$4.2_{1,43}$ (0.047)

Units for Garea and mass are mm^2 and g, respectively. Abbreviations: H, hard crayfish, HT total handling time; Lr, *Liodytes rigida;* Lp, *L. pygaea,* La, *L. alleni.* .RPA, relative prey area; S soft crayfish; ST, swallowing time.

TABLE S6 │ Final multiple regressions models predicting total handling time and the occurrence of some behaviors.

Model	Dependent Variable	Independent Variable	Standardized Coefficient	*p*
	L. rigida (*n* = 129)			
1	Log HT (s)	Log RPA	0.605	<0.0001
	(r^2 = 0.710)	If Envenomate	0.466	<0.0001
		H_S	0.366	<0.0001
2	Log swallow time	Log RPA	0.838	<0.0001
	(r^2 = 0.646)	H_S	0.294	<0.0001
		Coil	0.241	<0.0001
3	Coiling present	If Envenomate	0.653	0.001
	(r^2 = 0.298)	H_S	0.337	0.005
	L. pygaea (*n* = 27)			
4	Log HT(s)	Log RPA	1.135	0.0242
	(r^2 = 0.25)	Prey Type	-0.610	0.0095

TABLE S7 │ Univariate regressions predicting handling times and behavior for *L. pygaea* (n = 27).

**Independent Variable**	**Dependent Variable**	**Slope ± 95% CL**	**Intercept ± 95% CL**	r^2	p
Log RPA (18 fish)	log HT (s)	1.147 ± 1.087	0.254 ± 1.949	0.24	0.0398
Log RPA (9 siren)	log HT (s)	1.124 ± 2.401	0.903 ± 3.466	0.15	0.3048
Log RPA	Log Pre-Swallow Pause	0.827 ± 0.500	0.464 ± 0.842	0.96	0.0192
Log RPA	Log Swallow Time	0.938 ± 0.758	0.493 ± 1.277	0.21	0.0173
Log RPA	Strike Location	1.665 ± 1.230	-0.263 ± 2.072	0.24	0.0100

2 Supplementary Figures

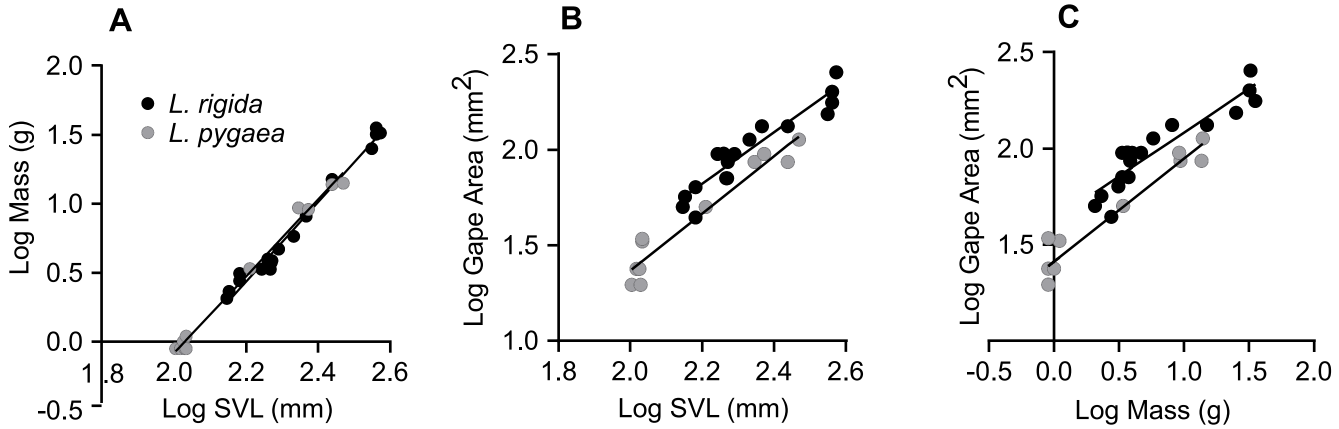

FIGURE S1 │ Scaling relationships for morphological data of *L. rigida* (*n* = 18) and *L. pygaea* (*n* = 11). (**A**) Mass versus SVL. (**B**) Maximal gape area versus SVL. (**C**) Maximal gape area versus mass. See supplementary tables S3 for regression statistics and S5 for ANCOVA results.

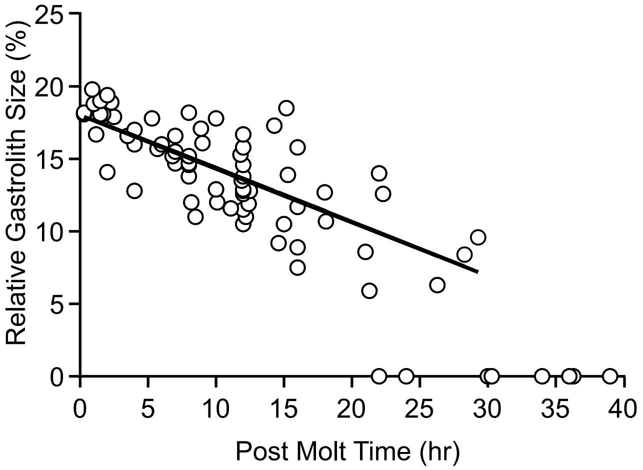

FIGURE S2 | Scaling relationship of relative gastrolith size versus time since crayfish molting (n = 78, Slope = -0.51±0.03, Y-intercept = 19.1±0.51, r^2=0.77, $p < 0.0001$). The relative gastrolith sizes of 0% were not included in the regressions because of the inability to know precisely when a value of 0 was attained.
